# Evaluation of bone mineral density and bone turnover in children on anticoagulation

**DOI:** 10.3389/fendo.2023.1192670

**Published:** 2023-08-01

**Authors:** Katharina Thom, Janina Maria Patsch, Florentina Haufler, Christiane Pees, Sulaima Albinni, Michael Weber, Christoph Male, Adalbert Raimann

**Affiliations:** ^1^ Division of Pediatric Cardiology and Haemostaseology, Department of Pediatric and Adolescent Medicine, Medical University of Vienna, Vienna, Austria; ^2^ Division of General and Pediatric Radiology, Department of Biomedical Imaging and Image Guided Therapy, Medical University of Vienna, Vienna, Austria; ^3^ Vienna Bone and Growth Center, Vienna, Austria; ^4^ Division of Pulmonology, Allergology and Endocrinology, Department of Pediatric and Adolescent Medicine, Medical University of Vienna, Vienna, Austria

**Keywords:** pediatric bone density, anticoagulation, chronic disease, osteoporosis, vitamin D

## Abstract

**Background:**

Childhood and adolescence are critical periods of bone mineral acquisition. Children on anticoagulation (AC) might have an increased risk for reduced bone mineral density (BMD). Risk factors for impaired bone accumulation include chronic diseases, immobility, and medication. Vitamin K (VK) deficiency reflected by undercarboxylated osteocalcin levels (ucOC) has been identified as a predictor of osteoporosis and fractures. Data on bone health in children under AC are sparse.

**Aims:**

To evaluate BMD in children on AC and characterize the risk factors of low BMD, including VK and Vitamin D (VD) status.

**Methods:**

Single-center cross-sectional study of clinical, biochemical, and densitometric parameters. Assessment of VK surrogate parameters included ucOC and matrix gla protein (MGP).

**Results:**

A total of 39 children (4–18 years; 12 females) receiving AC were included, 31 (79%) on VK antagonists and 8 (21%) on direct oral anticoagulants. Overall, BMD was decreased for both the lumbar spine (LS; −0.7SDS) and total body less head (TBLH; −1.32SDS) compared with pediatric reference data. Significant associations were found between early pubertal development and TBLH-BMD, and between BMI and LS-BMD. VK surrogate parameters were highly related to patients’ age and pubertal development. Neither serum parameters nor AC-related factors predicted BMD. VD was detected in 10/39 patients with lower values during puberty.

**Conclusion:**

Our data indicate BMD reduction in pediatric patients on AC. Although AC-related factors did not predict reduced BMD, low BMI and pubertal stages represented important risk factors. Awareness of risk factors for low BMD and high prevalence of VD deficiency during puberty could contribute to the improvement of bone health in this vulnerable patient group.

## 1 Introduction

Adequate bone mineral acquisition during childhood and adolescence is important for preventing fractures in children and young adults (1, 2). In recent decades, the frequency of children with chronic diseases (CD), such as congenital heart disease (CHD) and lung, kidney, and metabolic disorders, has increased because of improved medical and surgical treatment during childhood. In many of these patient groups, a reduction in bone mineral density (BMD) has been reported (3).

In adults, anticoagulation (AC) has been associated with secondary osteoporosis (4). Additionally, two investigations reported reduced BMD in children with congenital or acquired heart conditions (3, 5). A recent publication in children after Fontan palliation reported decreased BMD was most likely influenced by the underlying cardiac disease (6). Although these investigations on children with AC indicated an overall decreased BMD, the impact of underlying CD and long-term medication, such as AC, remains unclear. Additionally, there is a growing adolescent population with chronic diseases and sparse data concerning BMD and the vitamin D situation during pubertal stages (7, 8).

Vitamin K (VK) derivatives, such as phylloquinone (VK_1_) and menaquinone (VK_2_), represent essential cofactors for gamma-glutamyl carboxylase activity, regulating coagulation-protein function by posttranslational modification. Bone matrix proteins, such as osteocalcin (OC) and matrix Gla protein (MGP), also require VK-dependent carboxylation for adequate function. Direct and indirect effects of VK promoting osteoblast differentiation, function, mineralization, matrix acquisition, and Vitamin D (VD) metabolism have been reported (3, 5, 6). VK deficiency, as reflected by increased levels of undercarboxylated osteocalcin (ucOC), has been shown to be associated with osteoporosis and hip fracture (9).

Our study aims were to evaluate BMD in children on AC and to characterize risk factors of low BMD, including puberty stages and associations with serum parameters of VK and VD. To further characterize bone health in these patients, the objectives of the present study were to

- systematically assess BMD in children with CD on AC with VK antagonists (VKA) or direct oral anticoagulants (DOACs),
- investigate other potential influencing factors on BMD in patients with CD, with an emphasis on age and puberty,
- evaluate laboratory markers of bone turnover and surrogate parameters of VK status, and to assess VD status in this patient cohort.

## 2 Materials and methods

### 2.1 Study design and study population

In this cross-sectional study, children aged 1 to 18 years with CD on AC treatment at the Children’s Hospital, Medical University of Vienna, were eligible. Inclusion criteria were AC with VKA or DOACs for at least 3 months; eligible patients mainly had cardiac diseases or thrombophilia. Exclusion criteria were age <1 year and patients had to be clinically stable at the time of the study. The study was approved by the institutional ethics committee. Written informed consent was obtained from patients of appropriate age and their caretakers.

### 2.2 Study procedures

Details regarding diagnosis, medical history, current medication, and duration of AC were obtained from caretakers, patients, and medical records. Laboratory tests and bone densitometry were performed to screen for abnormal bone metabolism and low BMD.

### 2.3 Demographic parameters

Patients were clinically examined, and their pubertal stage was classified according to the Tanner scale (10). Anthropometric parameters were acquired in a standardized setting. Z-scores, including body mass index (BMI) and sitting height/leg length ratio as marker for body proportions, were calculated according to Austrian reference data (11). Documentation included self-reported history of bone pain and fractures/injuries, and family history of osteoporosis and fractures.

### 2.4 Bone densitometry

Bone densitometry was assessed using a Hologic QDR 4500 densitometer (Hologic, Bedford, MA). Measurements included lumbar spine (L1-L4), total body less head (TBLH), and bone mineral content. Z-scores were determined (12). Generally, BMD was compared with a historical group of healthy children and adjusted for height-and-age-Z-score (HAZ), which provides an adjustment of growth deficits according to the method described by Zemel et al. (13) Results were reported as standard deviation score (SDS). The official reference population for Hologic densitometers was used according to ICSDS recommendations (14). Decreased BMD was defined as an HAZ less than −2. Densitometry results were interpreted by a board-certified radiologist with experience in metabolic bone diseases and pediatric imaging.

### 2.5 Parameters of bone turnover and metabolism

Blood tests included serum electrolytes, alkaline phosphatase, iron status, and international normalized ratio (INR), reflecting the intensity of AC and VK-dependent activity of coagulation factors at the time of study inclusion.

VD status was assessed by serum 25OH-vitamin D_3_ (25(OH)D_3_) and classified in three groups: sufficient, >50 nmol/L; insufficient, 30–50 nmol/L; and deficient, <30 nmol/L (15). Analysis of parathyroid hormone levels and alkaline phosphatase activity as biochemical markers for rickets allowed the detection of skeletal response to VD deficiency, accordingly (16). C-telopeptide as a marker for bone resorption and turnover was assessed according to local standards (17). Regarding VK metabolism, the carboxylation of proteins involved in bone formation, such as OC, undercarboxylated OC (ucOC), and the dephosphorylated-uncarboxylated isoform of (dp-up) MGP, was investigated.

Standard laboratory testing was performed by the Department of Laboratory Medicine of the Medical University of Vienna. Blood for ucOC was separated by adsorption on hydroxyapatite prior to standard OC electrochemiluminescence-immunoassay analysis, as validated to allow the determination of a ucOC to total OC ratio (18). OC and plasma dp-uc MGP were analyzed using IDS-iSYS assays (IDS limited, UK) on an automated analyzer IDS-iSYS according to the manufacturer´s protocol. Measurements were performed at the Paediatric Endocrine Laboratory, Department of Paediatric and Adolescent Medicine, Medical University of Vienna.

### 2.6 Statistics

The sample size of this study was pragmatically based on the number of patients currently treated at our center that met the inclusion criteria. Data were processed using Jamovi version 2.2.5 (19). Categorical data were described using frequencies and percentages. For metric data, mean±SD (given normal distributions), or in the case of non-normal distribution median, first and third quartiles were reported. Shapiro–Wilk and Kolmogorov–Smirnov tests were used to check normality. Cohen’s d was assessed to indicate effect sizes. The one-sample Wilcoxon signed rank test was used to determine differences between the median of SDS-based parameters and the standard population median of 0 SDS. Depending on distribution, either Pearson’s or Spearman’s correlations coefficients were calculated to assess the linear association between metric risk factors and BMD. Additionally multiple linear regression models were adjusted for further factors, such as age and sex. To test for differences between two groups, either a) unpaired t-tests for metric, normally distributed data, and homogenous variances or b) Welch corrected t-tests in case of metric and normally distributed data with heterogenous variances, or c) Mann–Whitney U-tests for skewed data, were used. When comparing more than two groups, Welch’s corrected one-way ANOVA or Kruskal–Wallis tests were calculated, respectively. Effect size was calculated using Cohen’s d value (confidential intervals [CI] are indicated for 95%). A p-value of ≤ 0.05 was considered statistically significant.

## 3 Results

### 3.1 Study cohort

Thirty-nine patients aged 4–18 years (12 female) were recruited; two patients refused to participate. Nineteen patients were prepubertal, 15 were in the early pubertal stage and five were in the late pubertal stage. The most common underlying conditions were congenital or acquired cardiac diseases and thrombophilia.

The mean body height of the patients was reduced compared with an age matched Austrian reference population (−0.56 SDS, p=0.04). However, age-specific BMI SDS and body proportion SDS corresponded to the pediatric reference population (**Table 1**, **Figure S1**). Of the 39 patients, 31 (79%) were treated with VKA, 8 (21%) received DOACs, and 30/39 (77%) were receiving AC for >12 months (**Table 2**).

**Table 1:** Demographic and clinical characteristics.

Demographics		Median (range)	SD
Age (years)		13 (4-18)	7;15
Body length SDS		-0.62 (-3.1 – 1.7)	1.6;0.9
BMI SDS		0.21 (-4.9 – 3.4)	0.5;1.0
Body proportion SDS		0.463 (-4.4 – 3.2)	0.9;1.4
	**N (%)**		
Tanner			
Prepubertal (Tanner 1)	19 (49)	7y (4-17)	5;11y
Early pubertal (Tanner 2-3)	15 (38)	14y (12-18)	14;15y
Late pubertal (Tanner 4-5)	5 (13)	16.5y (13-18)	13-17y
Underlying diseases^a^			
Cardiac (congenital and acquired)^b^	27 (69)		
Cerebrovascular disorder^c^	5 (13)		
Thrombophilia^d^	11 (28)		
Autoimmune^e^	5 (13)		

^a^Nine patients had a combination of cerebrovascular disorders, thrombophilia, and autoimmune diseases. ^b^Inclusive of congenital heart disease and acquired heart disease (Kawasaki disease, cardiomyopathy, and mitral valve disease). ^c^Inclusive of ischemic strokes and cerebral-sinusvenous thrombosis. ^d^Antithrombin or protein c deficiency, homozygote factor V Leiden, and hypereosinophilia syndrome. ^e^Systemic lupus erythematodes, M. Behcet, and juvenile arthritis.

BMI, body mass index; SDS, standard deviation.

**Table 2:** Characteristics of anticoagulation (AC) therapy.

Type of anticoagulant	N (%)
VKA	31 (79)
DOAC	8 (21)
Duration of VKA therapy (months)	
3-6 months	4 (10)
6-12 months	5 (13)
>12 months	30 (77)
Duration of VKA therapy	60+/-49 (range 1-160)
**Duration of DOAC therapy (months)**	7.4+/-8.7 (range 1-26)
INR target	
2-3	24/31 (77)
2.5-3.5	7/31 (23)

DOAC, direct oral anticoagulants; VKA, vitamin k antagonists; INR, international normalized ratio.

### 3.2 Bone mineral density, puberty, and low BMI

**Figure 1:**
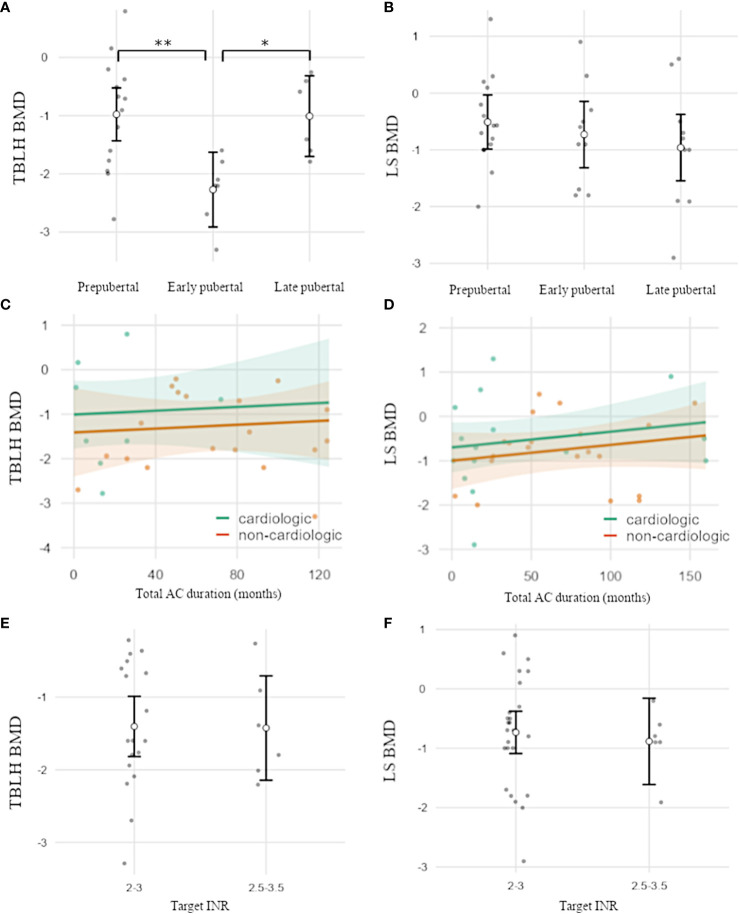
**(A–D)** BMD-HAZ according to pubertal development and the duration and intensity of AC. **(A)** TBLH-BMD was significantly reduced in early puberty versus prepubertal and late pubertal stages. **(B)** LS-BMD was not significantly associated with the pubertal stage and showed a trend toward lower BMD in later puberty. **(C–F)** AC duration and target INR were not associated with BMD. TBLH, total body less head; LS, lumbar spine; BMD, bone mineral density; AC, anticoagulation. Open circles indicate mean values. Error bars **(A, B, E, F)** indicate one standard deviation. Shaded areas **(C, D)** indicate a 95% confidence interval. Individual data points are shown as dots. *p<0.05; **p<0.01.

Mean BMD was below average for both locations (LS-BMD n=35: -0.7 SDS, p<0.001; TBLH-BMD n=27: −1.32 SDS, p<0.001), with significantly reduced values in patients under VKA treatment compared with the reference population. TBLH-BMD was significantly decreased in early puberty compared with prepubertal and late pubertal stages (overall/association pubertal stage: TBLH BMD p=0.006; early vs. prepubertal: p=0.007; early vs. late pubertal: p=0.029). LS-BMD showed a downward trend with increasing stages of puberty (**Figures 1A, B**).

Additionally, LS-BMD was strongly associated with BMI in spite of adjustments for age and sex but was not affected by other characteristics, such as height SDS or body proportions (p=0.006). For TBLH-BMD, a similar trend was observed but was not significant. (**Figure S2**, **Table S1**/analysis of covariance)

Neither duration or type of AC, cardiac vs. non-cardiac conditions, or target INR were associated with BMD (**Figures 1C–F**, **Table S2**/analysis of covariance). Nine of 39 patients (23%) reported at least one fracture of long bones (all trauma-associated), resulting in a fracture rate of 0.02 per patient year. Chronic bone pain was present in 29% of patients, with most of the visual analog scale scores between 0 and 5 and symptoms occurring approximately monthly. No positive family history for bone diseases were documented.

### 3.3 Carboxylation of osteocalcin and matrix Gla-protein

There were no associations between duration of AC or INR target and carboxylation status (**Table S3**). Mean total OC was 60.5 ±35 ng/dL, and ucOC showed levels of 59.4 ±17.0 ng/dL. Although ucOC levels revealed only moderate associations with patients’ age (R2 0.04), cOC was significantly decreased in older children (p=0.003, R2 = 0.21). Consequently, the ucOC:cOC balance shifted toward ucOC with pubertal development (p=0.003) and increasing age (p=0.001). For dp-uc MGP, an association with pubertal development was observed (p=0.01).

**Figure 2:**
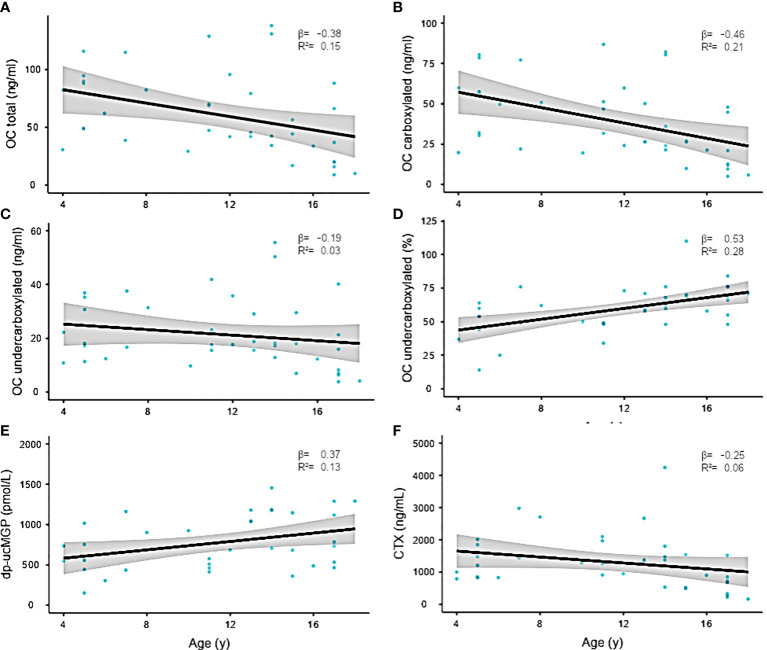
**(A–F)** Linear regression of serum bone parameters and age. Confidence interval (95%) indicated in gray. The regression coefficient is shown in the graph. Individual data points are shown as blue dots.

**Figure 3:**
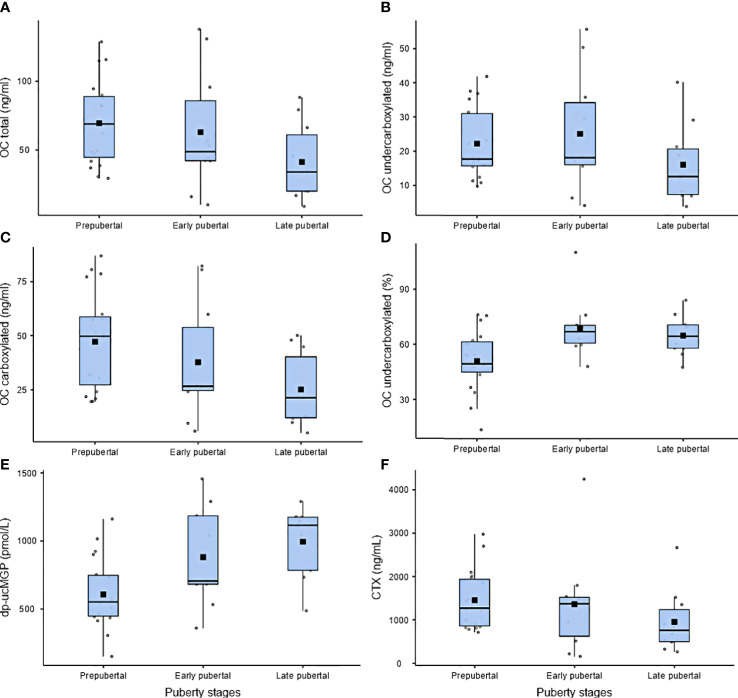
**(A–F)** Serum bone parameters according to pubertal development. Boxplots indicate first quartile, median, and third quartile. Error bars indicate maximum and minimum values. Solid squares indicate mean values. Individual data points are shown as gray dots.

To a lesser extent, dp-uc MGP revealed a positive correlation with age (p=0.045, R 0.37), while C-telopeptide as parameter for bone resorption and turnover remained relatively stable with pubertal development. All regression models were corrected for sex differences (**Figures 2**, **3**).

### 3.4 Vitamin D status

Ten (26%) children had VD deficiency, 12 (31%) showed VD insufficiency, and 14 revealed a sufficient 25(OH)D_3_ serum level above 50nmol/L. Lower VD levels were associated with puberty and increased BMI (p=0.035). Five of 12 patients with 25(OH)D_3_ insufficiency exhibited secondary hyperparathyroidism.

## 4 Discussion

The present study analyzed bone health parameters in patients on anticoagulation. We found decreased BMD levels, which were independent of type or duration of AC but associated with pubertal stages and BMI. Decreased VD levels and short stature further indicated the high vulnerability of bone health among these patients. The reduction of BMD is comparable to baseline levels in other pediatric disease cohorts, such as acute lymphatic leukaemia or inflammatory bowel disease (20). For the first time, we could demonstrate an association of pubertal development with decreased BMD. Interestingly LS-BMD and TBLH-BMD exhibited specific pubertal stages associated with low BMD.

Peak bone mineral accretion rates occur at around 12 years for girls and 14 years for boys (21). At 18 years of age, approximately 90% of peak bone mass has been achieved. In our cohort, especially in patients during early puberty stages, a reduced TBLH was observed. Most of our patients were so called “at risk” individuals with conditions associated with reduced bone mass and increased fracture risk. Two thirds of our patients had underlying cardiac diseases, mostly with reduced exercise tolerance. One third of patients had thrombophilia and were immobile as a result of ischemic strokes and cerebral palsy. These conditions potentially influenced BMD, as positive effects of, for example, high-impact low-frequency activity on bone health had been apparent in early puberty (22). The best activities for bone strength, such as walking, jogging, jumping, and dancing, were not common in our patients, which may also explain the reduced LS-BMD in the pubertal subgroups.

More than 20% of patients reported at least one (trauma associated) long-bone fracture. Although this is in line with pediatric data, the decreased mobility potentially affected risk behavior in our group and hampered direct comparisons (23, 24).

LS-BMD in our investigation was strongly associated with BMI but was not affected by other characteristics, such as height SDS, sex, or body proportions. Several studies also demonstrated higher incidences of osteoporosis in low-weight persons independent of sex and adolescent status (25–27). No patient in our cohort had a known eating disorder. However, similar effects may have occurred due to the underlying disease, e.g., growth retardation, unintended energy deficits or effects from chronic medication. The duration of AC or target INR did not show an association with BMD. A recent publication in patients after Fontan palliation reported decreased BMD that was most likely influenced by the medical condition. Patients on VKA showed a trend for decreased BMD (5, 28). Pediatric studies on osteoporosis naturally focused on pediatric populations with chronic diseases, such as CHD, lung disease, or kidney disease, under several medications. Available data indicate decreased BMD in these patient cohorts. However, the studies could not discriminate the influence and contribution of the underlying disease and medication on bone mineralization and decreased BMD (29).

Regarding serum parameters, we found a downward trend with age for total OC, which does not depict the entire complexity of pediatric OC reference ranges with peripubertal increase and postpubertal decrease (30). It is to be speculated whether this trend is due to the nature of linear regression models chosen for the given sample size or if the course of OC values differs in children and adolescents with chronic conditions. Although previous studies in adult patients have reported higher levels of ucOC with VKA than with DOAC, no differences were revealed in our pediatric cohort. However, previous studies in adult patients have reported higher levels of ucOC with VKA than with DOAC (31).

Vitamin K is necessary for the posttranslational modification by the VK-dependent carboxylase of bone matrix components, including OC and MGP. Carboxylated proteins have a high affinity for calcium and are important for the incorporation of calcium into the bone and supporting bone formation (31).

In healthy children, high levels of ucOC have been interpreted as poor VK status during bone growth. A peripubertal rise in the percentage of ucOC to cOC in our cohort with chronic conditions has been previously reported in healthy cohorts (32) Nevertheless, we did not observe associations of ucOC or dp-uc MGP with VKA-related factors or BMD. Our data suggest a highly age and puberty-dependent regulation of these surrogate parameters, impeding clinical usefulness in pediatric cohorts and chronic AC.

Our investigation revealed a high proportion of VD deficiency (26%) and insufficiency (31%). Pubertal patients had significantly lower 25D(OH)D_3_ values than prepubertal children. Measurements of serum VD reflect endogenous synthesis and dietary VD intake (33). Generally, there is limited evidence for recommending universal screening for VD deficiency. Our study population comprised an “at-risk” cohort potentially associated with reduced resorption, UV-B exposure, and mobility. VD supplementation has been shown to be effective in increasing BMD, particularly in adolescent girls (34).

Important limitations of our study include the small number of patients, which was due to the rare nature of the included cohort and the single-center study design. Reference data were derived from a historical group of healthy Austrian children. Furthermore, we only demonstrate observational characteristics and were not able to investigate the influence of other medications used by the cohort (e.g. steroids and diuretics).

## 5 Conclusions

Most of the investigated children on AC revealed reduced LS and TBLH-BMD. No association between AC, VK surrogate parameters, and BMD was found. However, we demonstrate a strong association between pubertal development and BMD reduction. BMD was decreased in patients with low BMI. A substantial proportion of our patients revealed VD deficiency. Awareness of risk factors for reduced BMD in this vulnerable patient group will have an impact on management regarding prophylaxis, nutritional, and physical activity recommendations.

## Data availability statement

The raw data supporting the conclusions of this article will be made available by the authors, without undue reservation.

## Ethics statement

The studies involving human participants were reviewed and approved by Ethics Committee of the Medical University of Vienna, Borschkegasse 8b/E06, 1090 Wien. Written informed consent to participate in this study was provided by the participants’ legal guardian/next of kin.

## Author contributions

All authors take full responsibility for data collection, data analysis, interpretation, and submission of the study. KT and AR conceived and designed the study, conducted analysis, and produced the tables. JP created and interpreted diagnostic reports. FH, CP, and SA contributed to data collection. AR conducted some of the laboratory analysis, produced the figures, and adapted tables. KT created the initial draft and edited and submitted the manuscript. CM carried out additional editing and advised on the analysis. All authors edited the manuscript and provided feedback on the study. All authors contributed to the article and approved the submitted version.
