# Supplementary material for: Evaluation of bone mineral density and bone turnover in children on anticoagulation
Source: Front Endocrinol (Lausanne). 2023 Aug 1;14:1192670. doi: 10.3389/fendo.2023.1192670 (PMC10433196; doi:10.3389/fendo.2023.1192670)
Supplement: Supplementary file 1 [file Table_1.pdf]

Evaluation of Bone Mineral Density and Bone turnover in children on anticoagulation

Analysis of Covariance - patient characteristics

Lumbar spine BMD					
	**Sum of Squares**	**df**	**Mean Square**	**F**	**p**
Age (y)	0.27	1	0.27	0.4	0.53
sex	0.23	1	0.23	0.3	0.56
BMI SDS	6.14	1	6.14	9.0	**0.006****
Puberty stages	0.72	2	0.36	0.5	0.60
Chronic condition	0.28	1	0.28	0.4	0.53
Residuals	19.12	28	0.68		

Total body less head BMD					
	**Sum of Squares**	**df**	**Mean Square**	**F**	**p**
Age (y)	0.34	1	0.34	0.5	0.48
sex	0.16	1	0.16	0.2	0.63
BMI SDS	1.8	1	1.8	2.7	0.11
Puberty stages	5.56	2	2.78	4.2	**0.029***
Chronic condition	0.72	1	0.72	1.1	0.31
Residuals	13.15	20	0.66		

Table S1 Patient characteristics - Analysis of Covariances. LS-BMD was strongly associated with BMI in spite of adjustment for age and sex but not affected by height SDS or body proportions (p=0.006). For TBLH-BMD a similar non-significant trend was found. LS-BMD: lumbar spine-bone mineral density, TBLH: total body less head, BMI: body mass index

Analysis of Covariance - treatment characteristics

Lumbar spine BMD					
	**Sum of Squares**	**df**	**Mean Square**	**F**	**p**
Oral anticoagulant treatment	0.05	1	0.05	0.1	0.80
total AC duration	1.28	1	1.28	1.5	0.23
Target INR	0.76	2	0.38	0.5	0.64
Residuals	25.19	30	0.84		

Total body less head BMD					
	**Sum of Squares**	**df**	**Mean Square**	**F**	**p**
Oral anticoagulant treatment	0.02	1	0.02	0.0	0.90
total AC duration	0.01	1	0.01	0.0	0.91
Target INR	0.84	2	0.42	0.4	0.67
Residuals	23.11	22	1.05		

Table S2 Treatment characteristics – Analysis of covariances: Neither duration or type of AC (anticoagulation), cardiac vs. non-cardiac conditions, or target INR were associated with BMD (bone mineral density).

Analysis of Covariance - Carboxylation status

	% undercarboxylated Osteocalcin				
	Sum of Squares	df	Mean Square	F	p
Oral anticoagulant treatment	588.54	1	588.54	2	0.17
Target INR	305.87	2	152.93	0.5	0.6
total time under AC (months)	125.24	1	125.24	0.4	0.52
Residuals	9706.2	33	294.13		

Table S3 Carboxylation status – Analysis of covariances: There were no associations between duration of AC (anticoagulation) or INR (international normalized ratio) target and carboxylation status
