# Supplementary material for: Evaluation of bone mineral density and bone turnover in children on anticoagulation
Source: Front Endocrinol (Lausanne). 2023 Aug 1;14:1192670. doi: 10.3389/fendo.2023.1192670 (PMC10433196; doi:10.3389/fendo.2023.1192670)
Supplement: Supplementary file 2 [file Image_1.pdf]

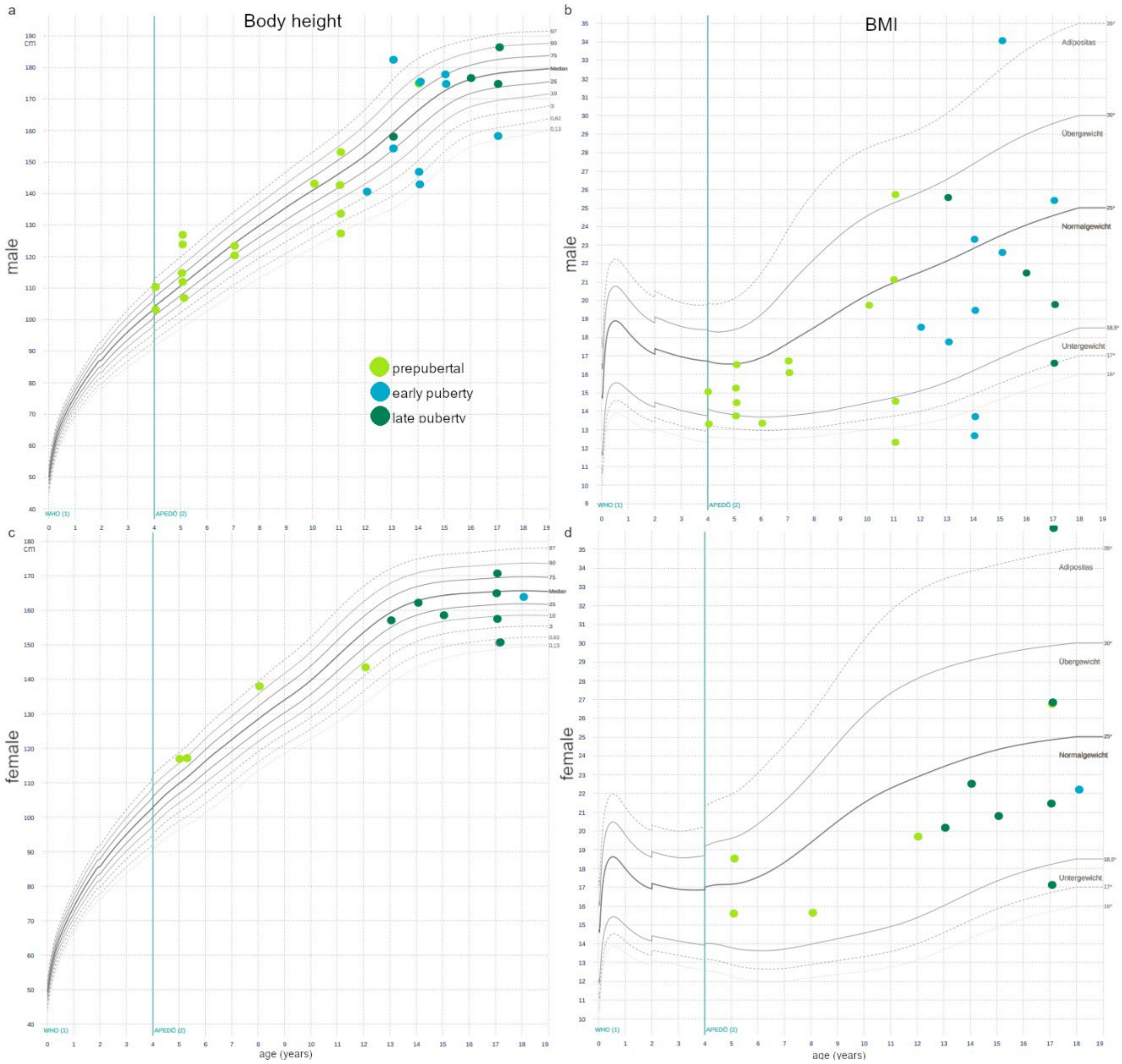

FIGURE S1. Body height (a, c) and BMI (b, d) of the study cohort marked on Austrian reference charts (11). Each dot represents a single patient. The pubertal stages are indicated by colors (light green, prepubertal; turquoise, early puberty; dark green, late puberty).

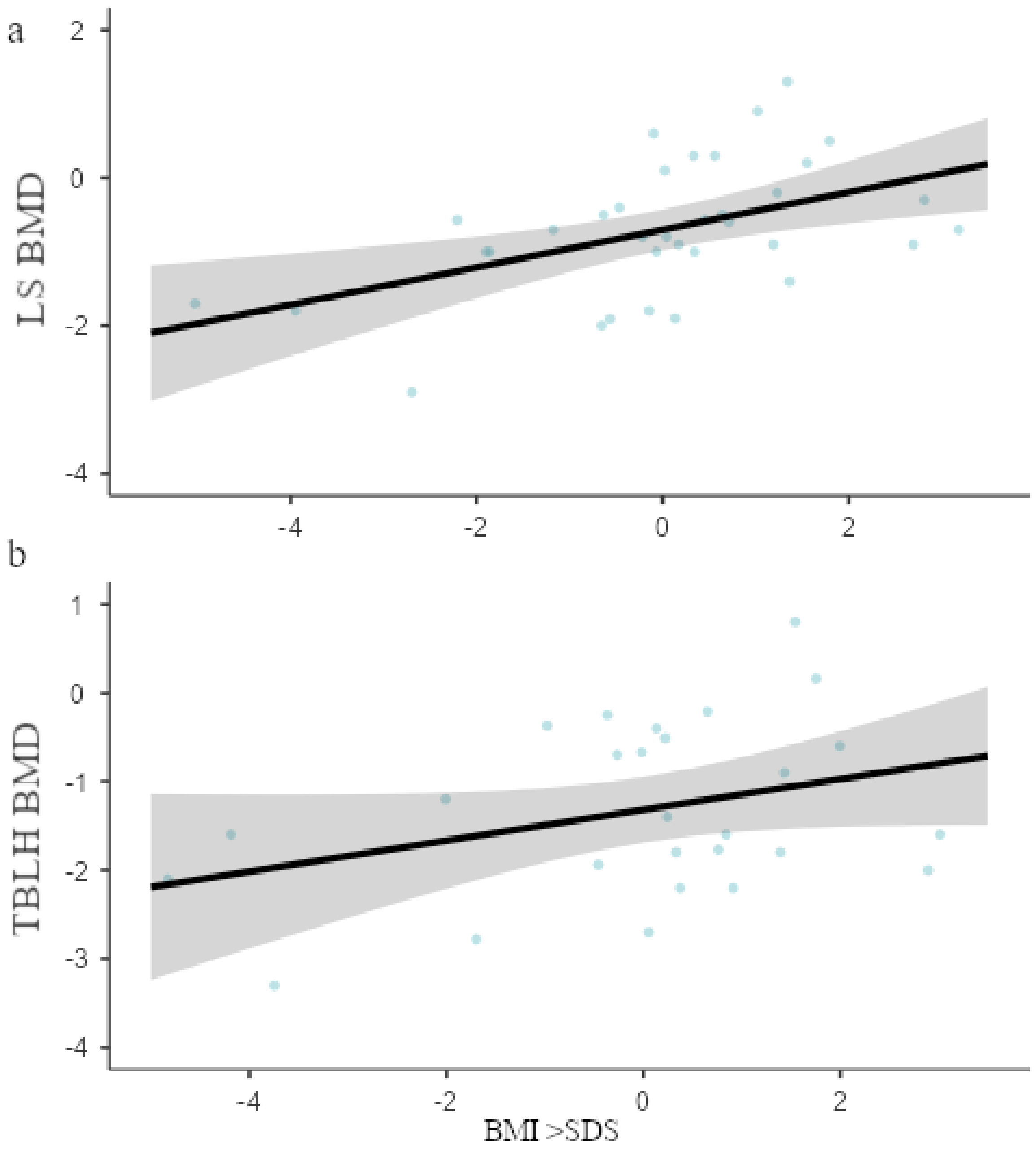

FIGURE S2. Linear regression of LS BMD (a) and TBLH BMD (b) height-adjusted Z-scores and BMI (body mass index) SDS. LS (lumbar spine) BMD (bone mineral density) was positively associated with BMI SDS (R=0.52; p=0.004). The confidence intervals (95%) are indicated by the gray areas. Individual data points are shown as blue dots.
